# Dietary biomarkers and food records indicate compliance to study diets in the ADIRA (Anti-inflammatory Diet In Rheumatoid Arthritis) trial

**DOI:** 10.3389/fnut.2023.1209787

**Published:** 2023-06-22

**Authors:** Anna Turesson Wadell, Linnea Bärebring, Erik Hulander, Inger Gjertsson, Rikard Landberg, Helen Lindqvist, Anna Winkvist

**Affiliations:** ^1^Department of Internal Medicine and Clinical Nutrition, Institute of Medicine, Sahlgrenska Academy, University of Gothenburg, Gothenburg, Sweden; ^2^Department of Gastroenterology and Hepatology, Clinical Nutrition Unit, Sahlgrenska University Hospital, Gothenburg, Sweden; ^3^Department of Rheumatology and Inflammation Research, Institute of Medicine, Sahlgrenska Academy, University of Gothenburg, Gothenburg, Sweden; ^4^Division of Food and Nutrition Science, Department of Life Sciences, Chalmers University of Technology, Gothenburg, Sweden

**Keywords:** diet, biomarkers, omega-3 fatty acids, whole grain, alkylresorcinols, carotenoids, dietary intervention

## Abstract

**Background:**

In the ADIRA (Anti-inflammatory Diet In Rheumatoid arthritis) trial, compliance to the study diets has previously been described primarily with a score based on reported intake of trial foods from telephone interviews. The aim of this study was to evaluate compliance using objective dietary biomarkers for whole grain, fruit and vegetables, margarine and oil, seafood and overall fat quality, as well as reported intake from food records of key components of the study diets.

**Methods:**

Fifty patients with rheumatoid arthritis were randomized to begin with the intervention diet (rich in whole grain, fruit and vegetables, margarine/oil and seafood) or the control diet (rich in meat and high-fat dairy) for 10 weeks, followed by a ~ 4 months wash-out period, and then switched diet. Compliance was evaluated using plasma alkylresorcinols (AR) as biomarkers for intake of whole grain wheat and rye, serum carotenoids for fruit and vegetables, plasma linoleic acid (LA, 18:2 n-6) and -α-linolenic acid (18:3, n-3) for margarine and cooking oil, plasma eicosapentaenoic acid (EPA, 20:5 n-3), −docosahexaenoic acid (DHA 22:6, n-3) and -docosapentaenoic acid (22:5 n-3) for seafood, and plasma fatty acid pattern for the overall dietary fat quality. Reported intake of whole grain, fruit, berries and vegetables, seafood, red meat, and fat quality was extracted from 3-d food records.

**Results:**

Plasma AR C21:0 and C23:0, LA, EPA, and DHA were higher while total serum carotenoids were lower after the intervention diet period compared to the control diet period (AR and carotenoids: *p* = <0.05, fatty acids: *p* = <0.001). Reported intake of whole grain, fruit, berries and vegetables, and seafood was higher and reported intake of red meat was lower during the intervention diet period compared to the control diet period (*p* = <0.001). Plasma- and reported fatty acid pattern differed as intended between the diet periods.

**Conclusion:**

This study indicates that the participants in the ADIRA trial were compliant to the study diets regarding intake of whole grain, cooking fat, seafood, and red meat, and the intended overall dietary fat quality. Compliance to instructions on fruit- and vegetable intake remains uncertain.

**Clinical trial registration:**

https://clinicaltrials.gov/ct2/show/NCT02941055?term=NCT02941055&draw=2&rank=1, NCT02941055.

## 1. Introduction

A dietary biomarker can be defined as a biological specimen that is an indicator of true dietary intake or the result of metabolism of dietary intake (1, 2). These may be used for validation of subjective dietary assessment tools or to measure compliance to dietary interventions (3). Biomarkers have the advantage of being an objective measure of intake in contrast to commonly used dietary assessments such as food frequency questionnaires (FFQ) and food records, which are merely subjective (4). Subjective assessments are prone to be biased by misreporting through, for example, underreporting of certain foods or lapses in memory (5). Different combinations of dietary biomarkers have previously been used to investigate compliance to interventions with whole diets such as a healthy Nordic diet (6, 7) and a Mediterranean diet (8–11). Although the ability to identify a dietary pattern through dietary biomarkers is uncertain, using dietary biomarkers to evaluate compliance to individual foods or food groups in controlled interventions is considered an appropriate approach (2).

Alkylresorcinols (AR) are phenolic lipids found in high amounts in the outer layer of wheat and rye among commonly consumed grains but in very low concentration in the starchy endosperm (12). Therefore, food items made of white flour contain almost no AR while whole grain products contain considerably more. Thus, AR have been evaluated as a biomarker for whole grain intake and are considered useful for this purpose (12–15).

Fruit and vegetables, especially those of yellow-orange color and dark green leaves, are the primary source of carotenoids in the diet (16). Although dairy products and eggs also contain low levels of these fat-soluble pigments, 80–90% of the carotenoid intake comes from the consumption of fruits and vegetables. Further, since the human body cannot synthesize carotenoids, they are commonly used as a biomarker of fruit- and vegetable intake (16, 17).

The two essential fatty acids linoleic acid (LA, 18:2, n-6) and α-linolenic acid (ALA, 18:3, n-3) are mostly found in vegetable oils and margarine, nuts and seeds (18). In the body, ALA can be elongated to eicosapentaenoic acid (EPA, 20:5, n-3), docosahexaenoic acid (DHA, 22:6, n-3) and docosapentaenoic acid (DPA, 22:5, n-3) but the formation of these longer fatty acids, especially DHA, is limited (19). Thus, intake of seafood, that is rich in EPA, DHA and DPA, correlates well with these fatty acids in plasma phospholipids (20). Also, interventions with a Mediterranean diet and with a healthy Nordic diet have shown alterations in plasma- and serum fatty acids resulting in a fatty acid composition with less saturated and more unsaturated fatty acids (21, 22), and in previous research on mussels- vs. meat intake there was a clear difference in the fatty acid pattern in plasma between the diets (23).

Rheumatoid arthritis (RA) is an autoimmune disease that primarily cause inflammation, and often destruction in the affected joints, although other organs may also be affected (24). Patients with RA often experience symptom relief or symptom worsening of specific foods, but, due to lack of evidence, there is no disease-specific dietary advice (25). ADIRA (Anti-inflammatory Diet In Rheumatoid Arthritis) was a randomized, controlled crossover trial evaluating the effects of a Mediterranean-like, proposed anti-inflammatory diet rich in whole grain, fruit and vegetables, and fatty fish on disease activity in RA. The trial was finished in 2018, and results on several outcomes have been published (26–31). So far, compliance to the ADIRA diets has been presented using a compliance scoring system including the study food items from a telephone interview and the nutrient intake from 3-d food records, which both indicated high compliance (26). Due to the inherent biases of these dietary assessments, there is a need to further investigate the compliance to the study diets in the ADIRA trial. The aim of this study was therefore to evaluate compliance by using objective dietary biomarkers. These included indicators of intake of whole grain wheat and rye (AR), fruit and vegetables (carotenoids), seafood (EPA, DHA and DPA) and margarine and oil (ALA and LA), and overall fat quality (plasma fatty acid pattern). Biomarkers were also compared with reported intake (3-d food records) of key components of the intervention, i.e., whole grain, fruit, berries and vegetables, seafood and red meat, and with the overall fat quality of the reported dietary intake. Finally, results for biomarkers and reported dietary intake were displayed for participants categorized as compliant vs. non-compliant according to the short scoring system.

## 2. Materials and methods

### 2.1. Study participants

Details on the study design and recruitment can be found elsewhere (26, 32). In brief, the ADIRA trial was a randomized, controlled crossover trial investigating the effects of a proposed anti-inflammatory diet on symptoms such as disease activity, health related quality of life and risk factors for cardiovascular diseases in patients with RA. It was performed at Sahlgrenska University Hospital, Gothenburg, Sweden in February 2017–May 2018. Participants were recruited mainly through the Swedish Rheumatology Quality Register (SRQ) and included patients 18–75 years old, with Disease Activity Score-28 (DAS28) ≥2.6 and a clinically stable disease as well as stable medication (i.e., no changes in immunosuppressive drugs preceding 8 weeks), without other serious illnesses, and who had the ability to understand the study information which was given in Swedish. Exclusion criteria were pregnancy or lactation, and allergies/intolerances to, or unwillingness to eat, the study food.

### 2.2. Study design

The participants were randomized to either the intervention- or the control diet for 10 weeks, followed by a wash-out period for a median (min-max) of 4 (2–5) months. After wash-out, participants switched to the other diet for 10 weeks. About 50% of their daily dietary intake (corresponding to ~1,100 kcal) for 5 days/week were provided by the study and delivered to their homes once a week during each diet period. During the study, the participants were told not to consume supplements other than those prescribed by their physician.

### 2.3. Study diets

The food items provided by the study are presented in detail in Supplementary Table S1. In brief, the intervention diet included low-fat dairy products with muesli or oatmeal, berries and a probiotic juice shot for breakfast, and two fruits (banana/apple/pear) as a daily snack. The main meal included either fish (2–4 times/week, mainly salmon) or legumes (1–2 times/week), potatoes, bulgur or whole grain cereal, and vegetables (e.g., spinach, snow peas, tomatoes, red pepper). During the intervention diet period, participants were instructed to limit red meat intake to ≤3 times/week, eat ≥5 portions of fruit and vegetables each day, use oil or margarine for cooking, and choose low-fat dairy and whole grain products.

The control diet included yoghurt and quark with corn flakes or white bread with butter and cheese, and orange juice for breakfast, and protein bar, −pudding or quark as a daily snack. The main meal included red meat or chicken, potatoes or rice, and a minimal amount of vegetables (e.g., canned tomatoes, haricot verts, onion). During the control diet period, the participants were instructed to eat red meat ≥5 times/week, limit fruit- and vegetable intake to ≤5 portions per day and seafood intake to ≤1 time/week, to use butter for cooking, choose high-fat dairy and to avoid probiotic products.

### 2.4. Compliance assessment

#### 2.4.1. Dietary biomarkers

**Figure 1:**
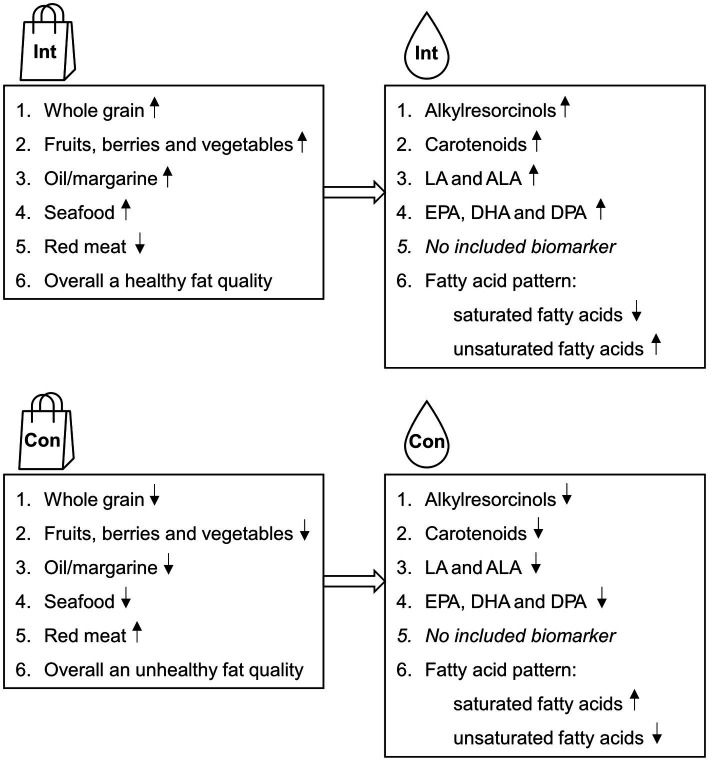
The expected changes in dietary intake (marked with a food bag) and the expected changes in the plasma/serum biomarkers (marked with a blood drop) during the intervention- and control diet periods in the ADIRA (Anti-inflammatory Diet In Rheumatoid Arthritis) trial. Up arrow = more of, down arrow = less of. ALA, α-linolenic acid; Con, Control diet; DHA, Docosahexaenoic acid; DPA, Docosapentaenoic acid; EPA, eicosapentaenoic acid; Int, Intervention diet; LA, Linoleic acid.

Plasma concentrations (nmol/L) of AR homologs C17:0, C19:0, C21:0, C23:0, and C25:0, the sum of these (“total alkylresorcinols”), and the ratio C17:0/C21:0, were used as markers for whole grain wheat and rye intake. The ratio C17:0/C21:0 indicates the source of the whole grain intake; rye or wheat (13). Serum concentrations (μmol/L) of carotenoids lutein, zeaxanthin, β-cryptoxanthin, lycopene, α-carotene and β-carotene, and the sum of these (“total carotenoids”), were used as markers for fruit and vegetable intake. In addition, due to previous research showing stronger associations with fruit and vegetable intake when excluding lycopene from total carotenoids (16, 33, 34), total carotenoids minus lycopene was also used as a marker for intake. Plasma concentrations of LA and ALA were used as markers for margarine- and oil intake and EPA, DHA, and DPA were used as markers for seafood intake (presented as % of total fatty acids). In addition, fatty acid patterns in plasma were used as a marker for differences in fat quality of the diets. Figure 1 shows the expected effects on both the intake and the biomarkers during the two diet periods.

#### 2.4.2. Reported dietary intake

The intake of whole grain, fruit, berries, vegetables, fatty acids, seafood, and red meat were calculated from 3-d food records performed in the end of each diet period. The instructions given to the participants on how to perform the food records have been described in detail previously (26). Most important, participants were instructed to weigh all food items consumed (except tap water) and if weighing was not possible, to use household measurements, or estimate portion size with help from pictures. They were also instructed to provide details on the type of fish/vegetable/fruit etc., and to record brand names when relevant. At each study visit, a dietitian reviewed the food record together with the participant. Food records were analyzed by the same dietitian using The Swedish Food Composition Database 2017-12-15 in Dietist Net Pro version 18.12.16 (Kost och Näringsdata AB). When certain food items were not available in the Swedish database, a Finnish version (Fineli 2018-02-28) or a food database providing nutrient information from the producers (2018-02-21) was used.

Daily intake of whole grain and fatty acids could be extracted directly from the database. However, daily intake of fruit, berries and vegetables, seafood, and red meat were manually extracted (see Supplementary Table S2). Since orange juice contains a high amount of β-cryptoxanthin (17) but was consumed daily during the control diet period, the additional fruit and vegetables-variable “Fruit, berries and vegetables incl. Juice and fruit-based beverages” (juice and other fruit-based beverages included in the main variable) was created.

### 2.5. The ADIRA-specific self-reported compliance scoring system

Results from the dietary biomarkers and the reported dietary intake were also compared with the self-reported compliance scoring system developed for the ADIRA study. Through a telephone interview performed once each diet period, consumption of provided study food during the previous week was captured. The participants were asked how much of each of the 15 meals that was consumed. Consumption of an entire meal equaled two points, part of the meal one point and if no parts of a meal were consumed, zero points. Altogether, this yielded a maximum of 30 points (3 meals x 5 days x 2 points) for each diet period, and 24 points (80%) were considered good compliance. As reported previously, 87% of control diet periods and 96% of intervention diet periods were completed with good compliance (26).

### 2.6. Laboratory analyses

All blood samples were collected after an overnight fast. Serum and plasma were separated through centrifugation at 2200 g for 10 min and 913 g for 10 min, respectively. Serum was analyzed by the central laboratory at Sahlgrenska University Hospital for C-reactive protein (CRP), erythrocyte sedimentation rate (ESR) and blood lipids. Serum and plasma were stored at −80°C until analysis of AR, carotenoids, and fatty acids.

Plasma AR concentrations (C17:0, C19:0, C21:0, C23:0 C25:0, their sum, and the ratio C17:0/C21:0) were measured by LC–MS/MS, as described elsewhere (35). Briefly, 100 μL of plasma was loaded on HybridSPEPlus phospholipid removal 96-well plates (Sigma-Aldrich) and ARs were recovered with acetone. After evaporating and resuspending the eluted samples in heptane-ethanol (95:5 volume/volume), extracts were transferred to chromatographic vials and analyzed by LC–MS/MS (QTRAP 6500+, AB SCIEX). Analytes were separated on a 50 × 2.1-mm amino column with 1.8-mm particles (Blue Orchid, Knauer) using a gradient program with solvents A and B; heptane and ethanol (99.7%), respectively. Ionization was conducted using atmospheric pressure chemical ionization in positive mode. Optimal conditions for the multiple reaction monitoring mode were set for individual AR homologs. The intra- and interbatch variations were <15% for all homologs.

For analysis of serum concentration of carotenoids, samples were thawed and 150 μL of serum was transferred to 1.5 mL polypropylene micro tubes and mixed with equal volumes of 95% ethanol containing 0.1% butylated hydroxytoluene. After mixing for 5 s on a vortex mixer at full speed, 500 μL of hexane was added and the samples were shaken vigorously for 5 min on a wibrax mixer (2,400 L/min). After centrifugation at 14,000 g for 2 min, 350 μL of the hexane phase was transferred to new 1.5 mL micro tubes before the hexane was evaporated with nitrogen gas. After 100 μL mobile phase (80% acetonitrile:20% methanol) had been added, samples were mixed for 2 min on a wibrax mixer set at 2,400 L/min followed by centrifugation at 14,000 g for 2 min. The analysis was performed with high-performance liquid chromatography (HPLC). Then, 20 μL of each sample was injected in a mobile phase that consisted of 80% acetonitrile and 20% methanol. The carotenoids were separated on a Chromolith Performance RP-18e, 100–2 mm column (Merck KGaA, Germany). The flow rate was 1.5 mL/min, and the used pump and detector were a PU 880 HPLC Pump and a Jasco MD-2010 Plus Multiwavelength Detector set at a wavelength of 450 nm (both from Jasco Inc., Japan). Signals were recorded using the software Clarity version 8.1.0.77 (DataApex Ltd., Czech Republic). Carotenoid concentrations were calculated from a standard curve generated from reference samples that were calibrated against an external standard from the National Institute of Standard and Technology (SRM 968f). Inter-assay coefficient of variations was less than or equal to 5. Zeaxanthin, a stereo isomer to lutein, could not be separated from lutein in the serum carotenoid analysis and therefore these are presented as only one value (“lutein + zeaxanthin”).

Analysis of fatty acids in plasma phospholipids has been described in detail previously (28, 36). In brief, total lipid was extracted into chloroform:methanol. Phosphatidylcholine was isolated by solid phase extraction and, through heating with methanolic sulphuric acid, fatty acid methyl esters were formed. These were separated by gas chromatography using a Hewlett Packard 6,890 gas chromatograph fitted with a BPX-70 column. By comparison with run times of authentic standards, fatty acid methyl esters were identified. All individual fatty acids are presented as % of total fatty acids.

### 2.7. Statistics

#### 2.7.1. Power calculation

The power calculation was based on the primary outcome of the ADIRA trial; DAS28. Thirty-eight participants were needed to be able to detect a difference of 0.6 in DAS28 with 90% power and the significance level set at 0.05.

#### 2.7.2. Statistical tests

Due to skewness in some data, all descriptive, continuous variables are presented as median (IQR). Categorical data are presented as number (%). Due to skewed distribution of the residuals for most AR- and carotenoid variables, all these variables were log10-transformed before analysis. The presented values are the transformed values.

To investigate compliance to the diets, a linear mixed ANCOVA model was used, including treatment (intervention or control diet), period (first or second diet period), sequence (order of the diets) and baseline value of the variable as fixed effects, and subject (the participant) as random effect. As AR and carotenoids are transported by lipoproteins in the body and therefore may be affected by triglyceride and cholesterol levels (33, 37, 38), and some carotenoids accumulate in low-density lipoprotein (LDL) rather than high-density lipoprotein (HDL) (39), triglycerides and total cholesterol were evaluated as possible confounders for AR and carotenoids, and LDL and HDL were evaluated as possible confounders for carotenoids. As inflammation has been shown to affect plasma and serum β-carotene levels (40), CRP was evaluated as a possible confounder for this carotenoid. Triglycerides and total cholesterol level exhibited confounding effects (a change in β-estimate of ≥10%) for total AR, the ratio C17:0/C21:0, the AR homologs C17:0, C19:0, C21:0, and C23:0, lutein + zeaxanthin, lycopene, β-carotene, and total carotenoids (triglycerides), and AR C17:0, lutein + zeaxanthin, β-carotene, and total carotenoids minus lycopene (total cholesterol). LDL and HDL exhibited confounding effects for lutein + zeaxanthin, β-carotene, total carotenoids and total carotenoids minus lycopene, and lutein + zeaxanthin, lycopene, β-carotene, total carotenoids, and total carotenoids minus lycopene, respectively. CRP exhibited no confounding effect for β-carotene.

Participants who completed at least one diet period were included in the main analyses on dietary biomarkers, and participants who completed at least one food record (during the intervention- or control diet period) were included in the main analyses on reported intake. In sensitivity analyses, diet periods where outcome variables deviated from normal distribution for the residuals or were considered outliers, were excluded.

To analyze fatty acid patterns (plasma and reported intake), Orthogonal Projections to Latent Structures (OPLS) were used to explore clustering patterns of observations, trends in the data in relation to known factors and outliers. OPLS models include not only x-values such as fatty acid data but also y-values, i.e., additional known factors that could influence the data. Y-values tested were body mass index (BMI), age, sex, ESR and a dietary quality index originally developed by the Swedish Food Agency (41) and adapted by Bärebring et al. (42). Presented OPLS-models only include y-values that have a CV-ANOVA *p* < 0.05 for the model. Separation of classes and variables related to separation in the data according to classification of diet (intervention diet vs. control diet) was evaluated using an OPLS with effect projections (OPLS-EP), comparing post values of each diet on paired samples using participants who completed both diet periods/food records. In addition, OPLS with Discriminant Analysis (OPLS-DA), where delta values for each period (post-pre) were used, was performed on participants who completed at least one diet period/food record. Cross-validation groups were set to standard (7:th sample) in OPLS-EP and OPLS but in OPLS-DA it was based on individual ID, so that both samples from one individual were left out in one cross-validation round. The validity of OPLS-DA models was assessed using permutation tests (*n* = 999). Validated prediction models for performance are presented using Receiver Operating Characteristics (ROC) Curve for OPLS-DA models. Cross-validated predictive residuals (CV-ANOVA) visual comparison between scores and cross-validated scores, the cumulative amount of explained variation in the data summarized by the model (R2X[*cum*] and R2Y[*cum*]), and the predictive ability of the model (Q2[*cum*]) are presented. Class discriminating fatty acids were regarded as most contributing if they were among those with the six highest VIP-scores among fatty acids with loadings w ≥ ± 0.1.

To evaluate the ADIRA-specific compliance scoring system, the median difference in post values (post intervention – post control) for plasma AR, serum carotenoids, plasma fatty acids (LA, ALA, EPA, DHA, and DPA) and the reported intake were visually displayed through bar charts by scoring status. Here, compliers were considered those participants who were defined as compliant to both diet periods.

The software IBM SPSS Statistics for Windows, version 29.0.0.0 (241) (Armonk, NY: IBM Corp.) was used for all statistical tests except tests regarding the fatty acid patterns which were performed using SIMCA software v.17.0 (Umetrics AB, Umeå, Sweden). A value of *p* of <0.05 was considered statistically significant.

### 2.8. Ethics approval and consent

The study was conducted in accordance with the Declaration of Helsinki. The regional ethical review board in Gothenburg (registration number 976-16, November 2016, and supplements T519-17, June 2017 and T878-17, October 2017) approved the study and all participants provided written and informed consent.

## 3. Results

### 3.1. Study participants

**Figure 2:**
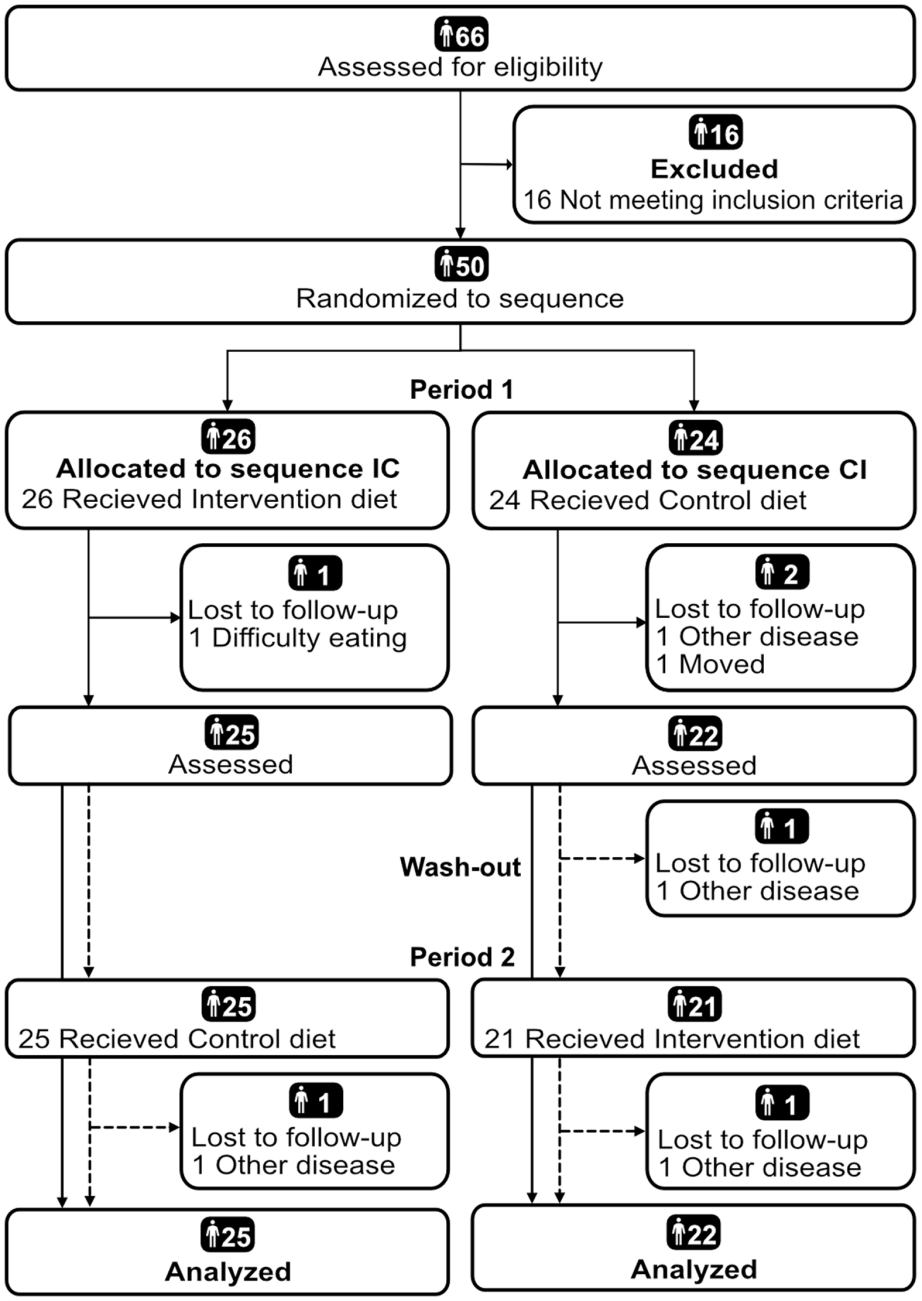
Flow chart diagram of participant inclusion in the ADIRA (Anti-inflammatory Diet In Rheumatoid Arthritis) trial reported according to CONSORT (43). Analyzed were participants who completed at least one diet period. CI, Control-Intervention; IC, Intervention-Control.

Of 66 screened patients, 16 were excluded due to remission defined as DAS28 < 2.6 (*n* = 14) or unstable disease (*n* = 2). Hence, 50 patients were included and randomized (Figure 2). Of the 47 participants that completed at least one diet period, almost 80% were women and median (IQR) age was 63 (54, 71) years old. Median (IQR) BMI was 27 (24, 32). Two participants (4%) were smokers. Table 1 describes the study population further. Forty-seven participants completed at least one diet period, while 44 participants completed both diet periods (Figure 2). As for the food records, 43 and 42 participants completed the 3-d food record during the intervention diet period and control diet period, respectively. Forty-six participants completed at least one food record during the diet periods and 39 participants completed both.

**Table 1:** Baseline characteristics of participants completing at least one diet period in the randomized crossover trial ADIRA (Anti-inflammatory Diet In Rheumatoid Arthritis).

	Baseline, screening/study visit 1 (*n* = 47)	Baseline, study visit 3 (*n* = 46)	Sequence IC (*n* = 25)^1^	Sequence CI (*n* = 22)^1^
Females, *n* (%)	36 (77)	36 (78)	20 (80)	16 (73)
Age (years)	62.8 (53.9, 70.8)	62.7 (53.1, 70.6)^1^	62.8 (59.3, 70.2)	64.3 (47.8, 72.4)
Education
Primary school	8 (17)	7 (15)^1^	4 (16)	4 (18)
Upper secondary school	16 (34)	16 (35)^1^	7 (28)	9 (41)
University degree or equal	23 (49)	23 (50)^1^	14 (56)	9 (41)
Occupational status
Does not work	20 (43)	20 (44)^1^	9 (36)	11 (50)
≤30 h/week	8 (17)	8 (17)^1^	5 (20)	3 (14)
≥30 h/week	19 (40)	18 (39)^1^	11 (44)	8 (36)
Current nicotine use
Cigarettes	2 (4)	2 (4)^1^	2 (8)	0 (0)
Snuff	4 (9)	4 (9)^1^	1 (4)	3 (14)
Other	2 (4)	2 (4)^1^	2 (8)	0 (0)
Anthropometrics
Body mass index (kg/m^2^)	26.6 (24.0, 31.8)	26.1 (23.1, 32.3)	26.9 (23.5, 33.2)	25.9 (24.1, 30.8)
Overweight (BMI 25.0–29.9), *n* (%)	14 (30)	15 (33)	7 (28)	7 (32)
Obese (BMI ≥30), *n* (%)	15 (32)	15 (33)	9 (36)	6 (27)
The rheumatic disease
Disease Activity Score-28	3.7 (3.0, 4.6)	3.3 (2.4, 4.4)	3.8 (3.1, 4.6)	3.3 (2.9, 4.6)
Erythrocyte sedimentation rate (mm)	20.0 (11.0, 26.0)	18.5 (8.8, 26.3)	20.0 (11.5, 26.5)	19.5 (8.8, 25.3)
C-reactive protein (mg/L)	3.0 (1.0, 6.0)	2.0 (1.0, 6.3)	3.0 (1.0, 5.0)	2.5 (1.0, 6.0)
Blood lipids
Triglycerides (mmol/L)	1.1 (0.8, 1.6)	1.1 (0.9, 1.5)	1.1 (1.0, 1.5)	1.1 (0.7, 1.6)
LDL-cholesterol (mmol/L)	3.3 (2.8, 4.2)	3.4 (2.8, 4.2)	3.4 (2.6, 4.1)	3.3 (3.0, 4.3)
HDL-cholesterol (mmol/L)	1.6 (1.3, 2.0)	1.7 (1.4, 2.0)	1.7 (1.5, 1.9)	1.5 (1.3, 2.2)
Total cholesterol (mmol/L)	5.5 (4.6, 6.0)	5.3 (4.7, 5.9)	5.4 (4.6, 6.0)	5.6 (4.6, 6.0)

Values refer to median (IQR) except when otherwise stated. ^1^Data collected at screening/study visit 1. BMI, Body mass index; CI, Control-Intervention; HDL, High density lipoprotein; IC, Intervention-Control; LDL, Low density lipoprotein.

### 3.2. Compliance

#### 3.2.1. Plasma alkylresorcinols, serum carotenoids, and plasma fatty acids

Total AR was higher after the intervention diet period compared to after the control diet period and this was borderline significant (mean difference: 0.125 nmol/L, 95% CI: −0.001, 0.251, *p* = 0.052, Table 2). The ratio C17:0/C21:0 was significantly lower after the intervention diet period compared to after the control diet period (mean: -0.185, 95% CI: −0.273, −0.097, *p* = <0.001). Among individual plasma AR homologs, C21:0 and C23:0 were significantly higher after the intervention diet period compared to after the control diet period (Table 2). Supplementary Table S3 shows the median (IQR) plasma AR concentrations on the original scale.

**Table 2:** Modeled estimates of differences in dietary biomarkers and dietary intake between the intervention- and the control diet period in the randomized crossover trial ADIRA (Anti-inflammatory Diet in Rheumatoid Arthritis).^1^

	Intervention		Control		Difference between periods^2^	95% CI	*p*
	Mean change	95% CI	Mean change	95% CI			
Alkylresorcinols
Total alkylresorcinols (nmol/L)^3,4^	0.072	−0.022, 0.166	−0.053	−0.146, 0.040	0.125	−0.001, 0.251	0.052
Ratio C17:0/C21:0^3,4^	−0.158	−0.244, −0.072	0.027	−0.058, 0.112	−0.185	−0.273, −0.097	<0.001
Alkylresorcinol homologs (nmol/L)
C17:0^3,5^	−0.054	−0.167, 0.059	−0.034	−0,145, 0.078	−0.020	−0.154, 0.114	0.765
C19:0^3,4^	0.033	−0.053, 0.118	−0.029	−0.113, 0.056	0.061	−0.046, 0.169	0.255
C21:0^3,4^	0.107	0.012, 0.202	−0.061	−0.155, 0.032	0.168	0.043, 0.293	0.010
C23:0^3,4^	0.096	−0.013, 0.205	−0.077	−0.184, 0.031	0.173	0.021, 0.325	0.027
C25:0^3^	0.041	−0.077, 0.159	−0.064	−0.180, 0.052	0.105	−0.057, 0.266	0.196
Carotenoids (μmol/L)
Lutein + zeaxanthin^3,6^	0.059	0.026, 0.092	0.048	0.016, 0.080	0.011	−0.037, 0.059	0.644
β-cryptoxanthin^3^	−0.018	−0.069, 0.033	0.113	0.063, 0.164	−0.131	−0.203, −0.059	<0.001
Lycopene^3,7^	−0.003	−0.044, 0.038	0.062	0.022, 0.102	−0.065	−0.123, −0.006	0.031
α-carotene^3^	−0.026	−0.081, 0.030	0.075	0.021, 0.130	−0.101	−0.179, −0.023	0.011
β-carotene^3,6^	−0.017	−0.061, 0.027	−0.003	−0.046, 0.041	−0.014	−0.078, 0.050	0.661
Total carotenoids (μmol/L)^3,8^	−0.006	−0.038, 0.025	0.045	0.014, 0.076	−0.052	−0.097, −0.006	0.026
Total carotenoids excl. Lycopene (μmol/L)^3,9^	−0.004	−0.037, 0.030	0.040	0.007, 0.073	−0.043	−0.091, 0.004	0.075
Fatty acids (% of total fatty acids)
Linoleic acid (18:2, n-6)	1.520	0.806, 2.234	−0.711	−1.414, −0.007	2.231	1.219, 3.243	<0.001
α-linolenic acid (18:3, n-3)	−0.003	−0.056, 0.050	−0.019	−0.071, 0.033	0.016	−0.049, 0.080	0.623
Eicosapentaenoic acid (20:5, n-3)	0.212	0.053, 0.370	−0.180	−0.336, −0.024	0.391	0.204, 0.579	<0.001
Docosahexaenoic acid (22:6, n-3)	0.406	0.273, 0.540	−0.272	−0.403, −0.141	0.678	0.491, 0.865	<0.001
Docosapentaenoic acid (22:5, n-3)	0.000	−0.020, 0.019	0.013	−0.007, 0.032	−0.013	−0.040, 0.014	0.327
Dietary intake (g/day)
Whole grain	8.71	3.15, 14.27	−15.76	−21.37, −10.15	24.47	16.57, 32.37	<0.001
Fruit, berries and vegetables	187.83	143.53, 232.13	−99.29	−143.99, −54.60	287.12	228.04, 346.20	<0.001
Fruit, berries and vegetables + juice	185.25	141.00, 229.51	15.99	−28.64, 60.61	169.27	108.20, 230.34	<0.001
Seafood	51.22	38.93, 63.50	−18.23	−30.65, −5.82	69.45	51.86, 87.04	<0.001
Red meat	−30.13	−39.97, −20.30	22.60	12.68, 32.53	−52.74	−65.13, −40.34	<0.001

^1^Plasma/serum: n = 47, dietary intake: *n* = 46. Dietary intake was assessed through 3-d food records. Analyzed by use of a linear mixed model with period, treatment, sequence, and baseline value as fixed effects and subject as random effect, except when otherwise stated. Intervention diet: a proposed anti-inflammatory diet rich in fatty fish, whole grain, fruits, and vegetables. Control diet: nutritionally alike a Swedish diet. ^2^Post intervention – post control. ^3^Log-10 transformed for normal distribution before analysis. The values presented are the transformed values. ^4^Analyzed by use of a linear mixed model with period, treatment, sequence, baseline value, and triglycerides as fixed effects and subject as random effect. ^5^Analyzed by use of a linear mixed model with period, treatment, sequence, baseline value, triglycerides and total cholesterol as fixed effects and subject as random effect. ^6^Analyzed by use of a linear mixed model with period, treatment, sequence, baseline value, triglycerides, total cholesterol, low-density lipoprotein, and high-density lipoprotein as fixed effects and subject as random effect. ^7^Analyzed by use of a linear mixed model with period, treatment, sequence, baseline value, triglycerides, and high-density lipoprotein as fixed effects and subject as random effect. ^8^Analyzed by use of a linear mixed model with period, treatment, sequence, baseline value, triglycerides, low-density lipoprotein, and high-density lipoprotein as fixed effects and subject as random effect. ^9^Analyzed by use of a linear mixed model with period, treatment, sequence, baseline value, total cholesterol, low-density lipoprotein, and high-density lipoprotein as fixed effects and subject as random effect.

Serum β-cryptoxanthin, lycopene and α-carotene were all significantly lower after the intervention diet period compared to after the control diet period (Table 2). The same was seen for total carotenoids with a mean difference (95% CI) of −0.052 (−0.097, −0.006) μmol/L (*p* = 0.026) between the diet periods. When excluding lycopene from total carotenoids, the difference was smaller and non-significant (mean: −0.043 μmol/L, 95% CI: −0.091, 0.004, *p* = 0.075). Supplementary Table S3 shows the median (IQR) serum concentrations of the carotenoids on the original scale.

Plasma LA, EPA, and DHA were all significantly higher after the intervention diet period compared to the control diet period (mean: 2.231, 95% CI: 1.219, 3.243, *p* = <0.001, mean: 0.391, 95% CI: 0.204, 0.579, *p* = <0.001, and mean: 0.678, 95% CI: 0.491, 0.865, *p* = <0.001, Table 2).

Sensitivity analyses excluding variables that deviated from the normal distribution with respect to the residuals or were considered outliers showed no major differences from the main analyses (data not shown).

#### 3.2.2. Reported dietary intake

The reported intake of whole grain, fruit, berries and vegetables (also when including juice and fruit-based beverages), and seafood was significantly higher after the intervention diet period compared to after the control diet period (mean [95% CI]: 24.47 [16.57, 32.37], 287.12 [228.04, 346.20], 169.27 [108.20, 230.34] and 69.45 [51.86, 87.04], respectively, *p* = <0.001; Table 2). The reported intake of red meat was significantly lower during the intervention diet compared to the control diet period (mean: −52.74, 95% CI: −65.13, −40.34, *p* = <0.001). Sensitivity analyses excluding variables that deviated from the normal distribution with respect to the residuals or were considered outliers showed no major differences from the main analyses (data not shown).

During the intervention diet period and the control diet period, 42 and 100% of the participants, respectively, reported following the study recommendations on the daily intake of fruit, berries and vegetables (assuming 1 portion = 100 g). For seafood, 67% of the participants reported following the recommendations during the control diet period, and for red meat 79 and 41% reported following the recommendations during the intervention and control diet period, respectively (assuming 1 portion = 125 g).

#### 3.2.3. The fatty acid patterns in plasma

**Figure 3:**
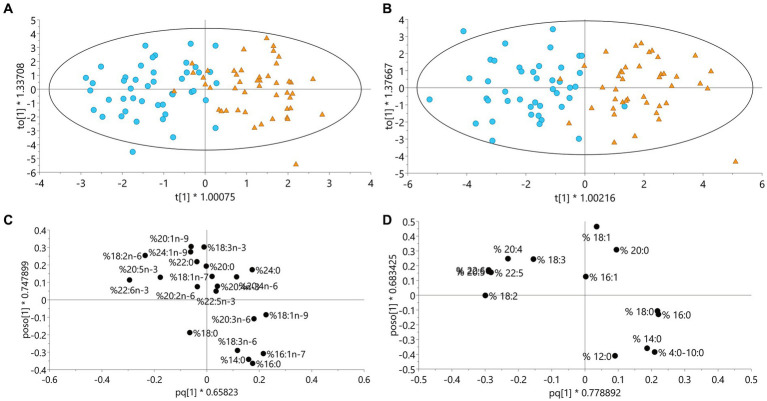
Models including data from all participants in the ADIRA (Anti-inflammatory Diet In Rheumatoid Arthritis) trial who completed at least one diet period. **(A)** OPLS-DA model for plasma fatty acids (%), *n* = 47, **(B)** OPLS-DA model for fatty acids (%) from dietary records, *n* = 46, blue circle = intervention, orange triangle = control. **(C)** Loadings for OPLS-DA model A (the fatty acids overlapping each other are 20:4 n-3 and 20:4 n-6), **(D)** Loadings for OPLS-DA model B (the fatty acids overlapping each other are 20:5 and 22:6), fatty acids to the left in figures **C** and **D** are associated with the intervention diet period and to the right with control diet period. OPLS-DA, Orthogonal Projections to Latent Structures with Discriminant Analysis.

In the OPLS model of fatty acid patterns in plasma (Table 3), age and BMI were included as y-values. Permutation tests confirmed that all OPLS-DA models had a sufficient quality. Most of the participants were correctly predicted to the correct diet period in the OPLS-DA model (Figure 3A). DHA (22:6 n-3) and LA (18:2 n-6) were among the most discriminating metabolites for the intervention diet and palmitic acid (16:0) and palmitoleic acid (16:1 n-7) were among the most discriminating metabolites for the control diet in both OPLS-DA and OPLS-EP models for plasma samples. EPA (20:5 n-3) also contributed to discriminate the intervention, and lignoceric acid (24:0), elaidic acid (18:1 n-9) and dihomo-gamma-linolenic acid (20:3, n-6) contributed to discriminate the control diet in the OPLS-DA model. Loadings for the OPLS-DA model is shown in Figure 3C.

**Table 3:** Model statistics of the fatty acid pattern in plasma and from 3-d food records in participants in the randomized crossover trial ADIRA ( Anti-inflammatory Diet In Rheumatoid Arthritis).

Model	Nr of Lv^1^	*N*	R2X [*cum*]^2^	R2Y [*cum*]^3^	Q2 [*cum*]^4^	CV-ANOVA^5^ (value of *p*)	ROC AUC^6^	Class (RA/K)	Permutation test (Q2)^7^
Plasma
OPLS baseline	2	47	0.210	0.297	0.210	Age *p* = 0.0062, BMI *p* = 0.0052			
OPLS-DA ΔI vs ΔC	1 + 5 + 0	91	0.581	0.677	0.483	2.2*10^−7^	0.98/0.98	89/88	−0.383
OPLS-EP ΔI vs ΔC	1 + 0 + 0	44	0.274	0.669	0.638	5.3*10^−10^			
Dietary intake
OPLS baseline	1 + 0 + 0	43	0.414	0.211	0.13	Age *p* = 0.062			
OPLS-DA ΔI vs ΔC	1 + 2 + 0	85	0.68	0.679	0.589	2.5*10^−13^	0.98/0.98	98/93	−0.181
OPLS-EP ΔI vs ΔC	1 + 0 + 0	39	0.605	0.674	0.649	3.8*10^−9^			

^1^Latent Variables. ^2^Cumulative fraction of the sum of squares of X explained by the selected latent variables. ^3^Cumulative fraction of the sum of squares of Y explained by the selected latent variables. ^4^Cumulative fraction of the sum of squares of Y predicted by the selected latent variables, estimated by cross validation. ^5^Analysis Of Variance testing of Cross-Validated predictive residuals. ^6^ROC AUC, Receiver Operating Curve Area under curve. ^7^The intercept between real and random models, degree of overfit. AUC, Area under the curve; C, Control; I, intervention; OPLS, Orthogonal Projections to Latent Structures; OPLS-DA, OPLS with Discriminant Analysis; OPLS-EP, OPLS with Effect Projections; ROC, Receiver Operating Characteristics.

#### 3.2.4. The fatty acid patterns from reported dietary intake

In the OPLS model of fatty acid patterns in diet (Table 3), none of the included y-values had a significant CV-ANOVA correlation, but age was borderline significant (*p* = 0.062). Most of the participants were predicted to the correct diet period in the OPLS-DA models based on fatty acids from the food records (Figure 3B). Figure 3D show loadings for the OPLS-DA model from food records. Increases in long chain omega-3 fatty acids and LA reflected the intervention diet and increases in palmitic acid reflected the control diet.

### 3.3. The ADIRA-specific self-reported compliance scoring system

In participants deemed compliant to both diets according to the ADIRA-specific self-reported compliance scoring system, the median differences between diets in total AR, C19:0, C21:0, lutein + zeaxanthin, β-cryptoxanthin, β-carotene, total carotenoids, total carotenoids minus lycopene, LA, ALA, EPA, DHA, and reported intake of whole grain and seafood, were more positive or less negative compared to in participants deemed to be non-compliant, i.e., consistent with the scoring system (Supplementary Figure S1). For DPA there was almost no difference between the diet periods and thus, no difference was detected between compliers and non-compliers.

## 4. Discussion

In this study, we investigated compliance to the study diets in the ADIRA trial using objective dietary biomarkers and compared these results with reported intake of key intervention foods from 3-d food records. Significant differences between the diet periods in plasma AR homologs, plasma fatty acids and reported intake indicated that participants were compliant regarding intake of whole grain, cooking fat, seafood, and meat. In addition, the fatty acid pattern in plasma as well as from reported intake also indicated compliance to the intended fat quality during the diets. Further, the food records displayed a significantly higher intake of fruit, berries and vegetables during the intervention diet period compared to the control diet period, indicating compliance also to these instructions. Contradictory, the level of serum carotenoids was lower after the intervention diet period compared to after the control diet period.

### 4.1. Whole grain

The reported intake of whole grain was significantly higher during the intervention diet period compared to the control diet period, indicating compliance. During the intervention diet, participants were instructed to choose whole grain products over refined products, but they were free to choose source of whole grain: wheat, rye, or oat, and no target amount was specified. The food provided by the study only included whole grain from primarily oat (oatmeal and muesli/granola daily) and also from wheat. This could explain the very small and only borderline significant differences between the diet periods in total AR and the significantly higher plasma concentrations of the individual homolog C21:0, predominant in wheat (44), after the intervention diet period compared to after the control diet period. Further, the ratio C17:0/C21:0 was lower after the intervention diet period compared to after the control diet period, indicating a higher intake of wheat rather than rye (13). Oat is not reflected by AR, which limits the possibility of this biomarker to capture changes in whole grain oat intake. Unfortunately, no established biomarker for oat or whole grain oat exists, although avenanthramides and avenacosides are promising and under current investigations (45).

### 4.2. Fruit, berries, and vegetables

Unexpectedly, the serum levels of total carotenoids, β-cryptoxanthin, lycopene and α-carotene were significantly lower after the intervention diet period compared to after the control diet period. This contrasts with the reported dietary intake, which showed a significantly higher intake of fruit, berries and vegetables during the intervention diet period compared to during the control diet period. Although carotenoids generally are considered appropriate biomarkers for total fruit- and vegetable intake, the concentration and type of carotenoids differ in different types of fruit and vegetables. Thus, the choice of fruit and vegetables influence the accuracy of carotenoids as a biomarker. Much of the vegetables provided the participants during the intervention diet period, e.g., baby spinach, ruccola and carrot, are rich in carotenoids (17). However, the fruit provided were apples, pears, and bananas which in contrast to, e.g., watermelon, papaya, and oranges, are low in carotenoids.

During the control diet period participants were provided with orange juice to consume 5 days/week. Orange juice contains a high amount of the carotenoid β-cryptoxanthin and is one of the foods contributing the most to total intake of β-cryptoxanthin in several European countries (17). To examine this further, we added juice to total fruit- and vegetable intake and could see a smaller difference between the diet periods and there was no longer a significant reduction during control period, but rather a non-significant increase. Thus, juice intake could be an explanation for the unexpected results for β-cryptoxanthin. In addition, the control diet was rich in meals based on canned or pureed tomatoes, which are rich in the carotenoid lycopene. A 2015 meta-analysis (46) showed that only lutein, b-cryptoxanthin, a-carotene and b-carotene were considered appropriate biomarkers of intake of fruit and vegetable intake, while lycopene was not. In addition, Jansen et al. (16) reported lycopene to contribute most to total carotenoid levels while it was the only carotenoid not positively correlated to fruit and vegetable intake. Further, when lycopene was excluded from the sum of carotenoids, total carotenoids was more strongly correlated with fruit and vegetable intake (16). Others have reported associations between lycopene and vegetable intake only in unadjusted analyses (33) or not at all (34). For this reason, we also performed an analysis where lycopene was removed from total carotenoids. Here, the difference between the diet periods was still in the opposite direction to that expected, but now smaller and non-significant.

Finally, we instructed participants to consume ≥5 portions/day of fruit, berries and vegetables during the intervention diet period, and ≤ 5 portions/day during the control diet period. This means that 5 portions/day were allowed during both diet periods. Although the reported dietary intake shows a higher consumption during the intervention diet period compared to during the control diet period, the food records may have been biased through misreporting, e.g., through a wish to report what was believed to be the” correct” intake during each diet period (47, 48).

### 4.3. Fatty acid pattern – cooking fat, seafood, and meat

As expected, plasma LA, EPA, and DHA increased significantly during the intervention diet period. Further, the plasma fatty acid pattern showed a clear separation between the two diet periods with the polyunsaturated LA, EPA, and DHA being the most discriminating fatty acids for the intervention diet period, and the saturated palmitic acid and lignoceric acid being some of the most discriminating fatty acids for the control diet period. Further, almost all individuals were classified into the correct diet period regarding both plasma fatty acid pattern as well as the fatty acid pattern from the reported intake. In addition, reported intake of seafood was considerably higher during the intervention diet period compared to the control diet period. These results all indicate compliance to the intended fat quality during both diet periods.

More surprisingly, no significant difference in plasma ALA could be seen between the diet periods. The intervention diet was rich in rapeseed oil and walnuts, which are some of the food items containing the most ALA (49). However, the control diet included several ready meals containing rapeseed oil as well as a butter-based sandwich spread that also contained some rapeseed oil. In addition, several studies have shown no correlation between ALA intake and ALA levels in plasma- or serum phospholipids (6, 50, 51). Since the different blood fractions differ in their fatty acid composition (20), perhaps our results would have been different if ALA in, e.g., total plasma or cholesteryl esters had been used instead.

Positive correlations between fish intake and plasma EPA, DHA and DPA have been previously observed (52). Even so, plasma EPA and DHA may not be suitable biomarkers for fish intake but rather for EPA- and DHA intake (4). In previous research from our group, modeling of several fatty acids was a better biomarker for seafood intake than was individual fatty acids (23). In the current study, plasma EPA and -DHA differed significantly between the diet periods in the same direction as the reported seafood intake. This is probably due to the choice of seafood in our intervention diet: almost exclusively salmon, which is rich in these n-3 fatty acids. If leaner seafood had been consumed, the results may have been different (53).

As desired, the reported consumption of red meat was significantly lower during the intervention diet period compared to the control diet period. To some extent, the difference is supported by the fatty acid pattern. Compared to fatty fish which has a higher proportion of polyunsaturated fatty acids, meat contains more saturated than polyunsaturated fatty acids (49). Further, palmitic acid and palmitoleic acid were among the most discriminating metabolites for the control diet, and these are two of the major fatty acids in red meat (54). Unfortunately, there are currently no established biomarkers for red meat. Urea nitrogen, creatin, creatinine and carnosine are all examples of metabolites that have been proposed but are not specific for meat intake (55). Further, it seems that our participants found it easier to follow the recommendations of low intake of red meat during the intervention diet period compared to the high intake during the control diet period, indicated by higher compliance to red meat recommendations during the intervention diet period. Patients with RA have reported experiencing symptom worsening from eating red meat (25) and often avoid meat in their diet (56). Compared to the average red meat intake of 67 g/day in the Swedish adult population (57), our study population consumed little meat at baseline (46–49 g/day) and perhaps they avoided increasing intake during the control diet period. We did not give instructions on exact amount of meat to consume during the diet periods. Thus, participants with small appetite or lower energy needs may have consumed 5 portions of meat per week, but less than 125 g per portion, and therefore were miscategorized as not following the instructions.

### 4.4. The ADIRA-specific self-reported compliance scoring system

The ADIRA-specific self-reported compliance scoring system has been used to describe compliance to the study diets in several previous publications (26–31). The scoring system estimated good compliance (26), and this conclusion is supported by the overall results of the dietary biomarkers and food records reported here. We wanted to compare the results of the scoring system with the objective dietary biomarkers and the food records which captured the whole intake, since the scoring system was merely subjective and only included consumption of the foods provided by the study. Further, we believed this analysis to be valuable since a simple yet valid way to measure compliance in intervention studies is desirable. In compliers (according to the scoring system), the differences between the diets in most biomarkers and reported intake of whole grain and seafood, were more positive compared to in non-compliers. This means that the result of the scoring system was consistent with the results of most biomarkers and of the reported intake of these foods, i.e., the scoring system captured compliance quite well.

### 4.5. Compliance to dietary interventions in general

Compliance to a dietary intervention may be challenging for the participant. Sherman et al. list several factors that may affect compliance unique for dietary interventions in contrast to, e.g., pharmacological: decision making, social and cultural contexts, perceptions, preferences and environmental factors (58). In the grocery store, the number of food items is countless, and knowledge is required when making choices. Further, religion may impact dietary behaviors and thus affect the desired change. The individual’s taste and economic situation are of course also possible reasons for lack of compliance in dietary interventions. Still, compliance is vital for correct analyses of the outcomes studied (6, 59). Adherence to the ADIRA study diets may have been challenging for some of the participants, but this study indicates that they succeeded. One reason for that could be that the participants were provided with study food delivered to their homes, and with menus and recipes. There are several examples of whole diet interventions where food has been provided to the participants and that report high compliance (60–63). Further, the communicated possible reduction of the RA symptoms was of course motivating, and since we tried to blind the participants by not communicating which of the diets was the intervention- and which was the control diet, this was possibly motivating to also follow the control diet.

### 4.6. Strengths and limitations

There are both study limitations and -strengths to discuss. First, the crossover design with a long wash-out period is a strength. Because each participant is his/her own control when a crossover design is used, possible confounding effects were minimized. Further, by providing the participants with foods and recipes for 5 days/week (approximately 50% of their intake) and easy-to-cook- or ready meals, and by using home delivery, the pre-requisites for high compliance were good. It may have been even better if food and recipes for 100% of intake had been provided. However, if participants would have been unable to cook some of their own familiar dishes during the trial it may have resulted in more dropouts. The dietary biomarkers included here have some limitations for use in the ADIRA study. Still, by using objective dietary biomarkers to measure compliance, the results are less biased by misreporting than the results from more subjective assessments. Social desirability or social approval may have affected the participants’ reporting of food intake in the food records (64). Yet, by using several different biomarkers, and food records as a complement, we believe that we managed to capture compliance to almost all of the key components of the diets. With this study we showed the need to complement established dietary biomarkers with other dietary assessments when evaluating compliance to dietary intervention studies, depending on the source of whole grain and fruit and vegetables. Finally, the results of our study not only show high compliance regarding the intake of whole grain, cooking fat, seafood and red meat, and thus validate the results of our previous studies from the ADIRA trial, but they also indicate feasibility of the intervention diet.

## 5. Conclusion

Dietary biomarkers and food records indicate that participants in the ADIRA trial were compliant to the study diets regarding intake of whole grain, cooking fat, seafood, and red meat, and the intended overall dietary fat quality. The reported intake of fruit, berries and vegetables were higher during the intervention diet period compared to the control diet period, but this was not supported by serum carotenoids. Thus, study compliance to instructions on fruit and vegetable intake remains uncertain.

## Data availability statement

The raw data supporting the conclusions of this article will be made available by the authors, without undue reservation.

## Ethics statement

The studies involving human participants were reviewed and approved by the regional ethical review board in Gothenburg, Sweden. The patients/participants provided their written informed consent to participate in this study.

## Author contributions

AW, HL, LB, and IG designed the study. ATW, LB, HL, AW, and EH conducted the research. RL performed the analyses of plasma alkylresorcinols. HL performed the statistics on the fatty acid patterns. ATW performed all other statistics, wrote the manuscript, and had primary responsibility for the final content. All authors assisted in interpreting the data and have read and approved the final manuscript.

## Funding

This study was funded by the Swedish government under the ALF agreement (ALFGBG-74630), Swedish Research Council for Health, Working Life and Welfare (FORTE, https://forte.se/en/), the Lennander Foundation (Sahlgrenska University Hospital Foundations), the Inger Bendix Foundation (https://www.Stiftelsemedel.se/inger-bendix-stiftelse-fr-medicinsk-forskning/), and the Gothenburg Region Foundation for Rheumatology Research (GSFR). The Gothenburg University Library provided a discount on the publication fee. The funders had no role in the design of the study and collection, analysis, and interpretation of data and in writing the manuscript.

## Conflict of interest

The authors declare that the research was conducted in the absence of any commercial or financial relationships that could be construed as a potential conflict of interest.

## Publisher’s note

All claims expressed in this article are solely those of the authors and do not necessarily represent those of their affiliated organizations, or those of the publisher, the editors and the reviewers. Any product that may be evaluated in this article, or claim that may be made by its manufacturer, is not guaranteed or endorsed by the publisher.

## Abbreviations

ADIRA: Anti-inflammatory Diet In Rheumatoid Arthritis
AR: Alkylresorcinols
DAS28: Disease Activity Score-28
RA: Rheumatoid Arthritis
