# Supplementary material for: Dietary biomarkers and food records indicate compliance to study diets in the ADIRA (Anti-inflammatory Diet In Rheumatoid Arthritis) trial
Source: Front Nutr. 2023 Jun 22;10:1209787. doi: 10.3389/fnut.2023.1209787 (PMC10325030; doi:10.3389/fnut.2023.1209787)
Supplement: Supplementary file 1 [file Data_Sheet_1.PDF]

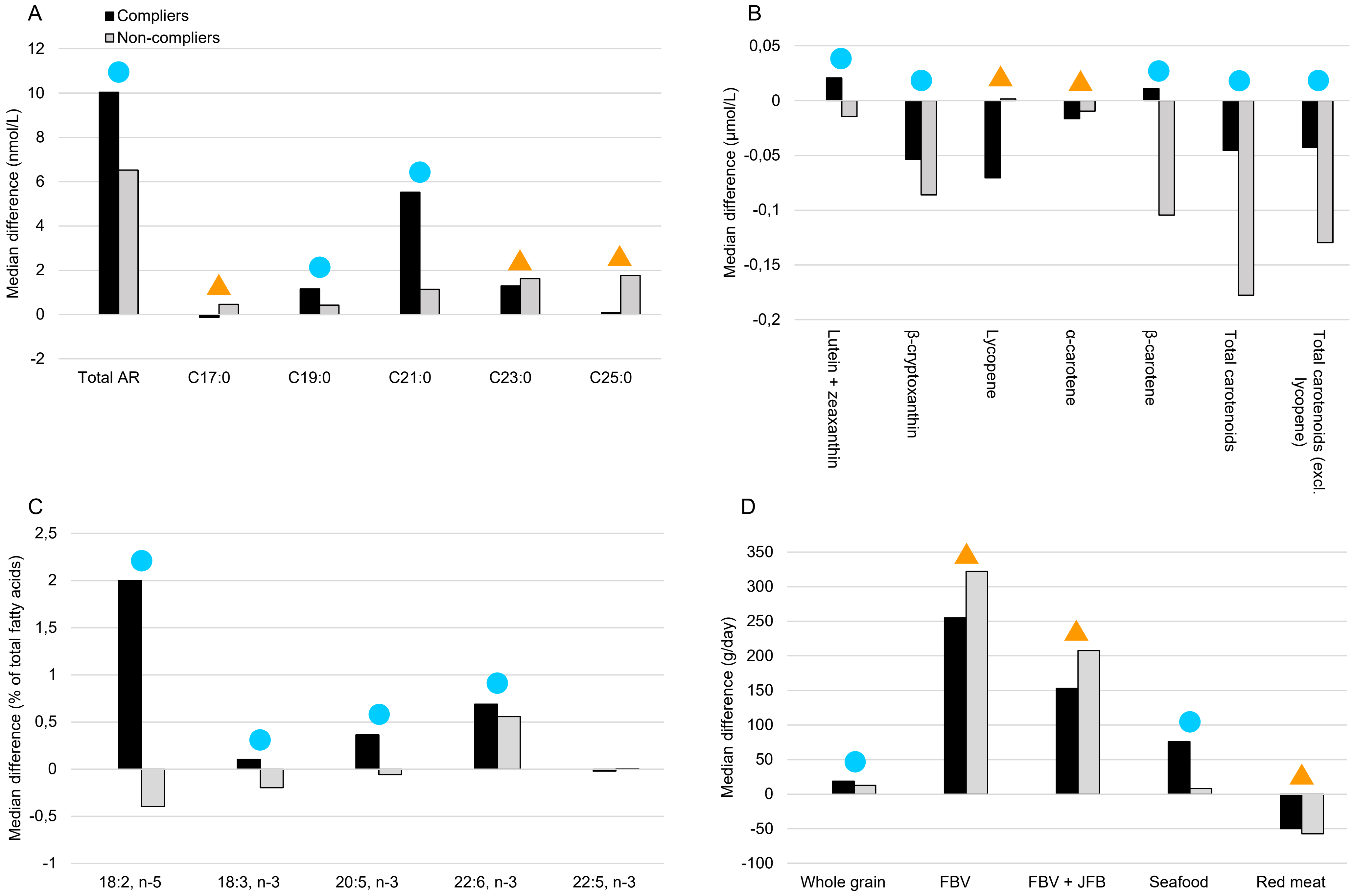
A
Compliers
Non-compliers
Median difference (nmol/L)
12
10
8
6
4
2
0
-2
Total AR
C17:0
C19:0
C21:0
C23:0
C25:0
B
Median difference (µmol/L)
0,05
0
-0,05
-0,1
-0,15
-0,2
Lutein + zeaxanthin
β-cryptoxanthin
Lycopene
α-carotene
β-carotene
Total carotenoids
Total carotenoids (excl. lycopene)
C
Median difference (% of total fatty acids)
2,5
2
1,5
1
0,5
0
-0,5
-1
18:2, n-5
18:3, n-3
20:5, n-3
22:6, n-3
22:5, n-3
D
Median difference (g/day)
350
300
250
200
150
100
50
0
-50
-100
Whole grain
FBV
FBV + JFB
Seafood
Red meat

SUPPLEMENTARY FIGURE 1| Median difference in dietary biomarkers and reported dietary intake between post intervention- and post control diet periods ($intervention - control$), clustered by compliance[1] to the diets. A) Plasma alkylresorcinols (nmol/L), B) Serum carotenoids (µmol/L), C) Plasma fatty acids (% of total fatty acids) and D) Reported dietary intake (g/day) of whole grain, fruit, berries and vegetables, fruit, berries and vegetables incl. juice and fruit-based beverages, seafood, and red meat. Blue circle = Results are consistent with results from the study-specific compliance scoring system, Orange triangle = Results are *not* consistent with results from the study-specific compliance scoring system

[1]Measured using the objective ADIRA-specific scoring system. Compliers=participants compliant to *both* diets. Compliers (plasma/serum): n=36, non-compliers: n=8, compliers (dietary intake): n=32, non-compliers: n=7. ADIRA, Anti-inflammatory Diet In Rheumatoid Arthritis; AR, Alkylresorcinols; FBV, Fruit, berries and vegetables; JFB, Juice and fruit-based beverages
