# Supplementary material for: Dietary biomarkers and food records indicate compliance to study diets in the ADIRA (Anti-inflammatory Diet In Rheumatoid Arthritis) trial
Source: Front Nutr. 2023 Jun 22;10:1209787. doi: 10.3389/fnut.2023.1209787 (PMC10325030; doi:10.3389/fnut.2023.1209787)
Supplement: Supplementary file 2 [file Data_Sheet_2.PDF]

Supplementary Material

SUPPLEMENTARY TABLE 1 | Details of the ADIRA diets

			Number of meals	Comment
**Intervention diet**	**Breakfast**	Low-fat milk or sour milk	50	
		Oatmeal	20	
		Muesli/granola (whole grain)	30	
		Walnuts	35	
		Blueberries or pomegranate	50	
		Probiotic shot	50	
	**Main meal**			
	Carbohydrate	Wheat berries (whole grain)	12	
		Bulgur	11	
		Potatoes	21	
		Pasta (whole grain)	6	
	Protein	Salmon	38	Burgers including both salmon and cod (8 meals)
		Legumes	12	
	Greens	Spinach	9	
		Ruccola	22	

		Number of meals	Comment
	Edamame beans	14	
	Snow peas	6	
	Green peas	12	
	Broccoli	8	
	Tomatoes	6	Fresh/canned
	Cabbage	3	
	Carrot	10	
	Parsnip	4	
	Pepper	9	
	Fennel	6	
	Mushrooms	3	
	Mango	8	
	Apricots	3	
	Green onions/onion/garlic	50	
Sauce	Coconut milk	3	
	Yoghurt	14	
	Cream	8	
	Low-fat Crème Fraîche	12	
Other	Flavoring: Soy sauce, broth, lime juice, ginger, spices etc.	50	
	Rapeseed oil	22	

			Number of meals	Comment
		Sunflower oil	7	
		Olive oil	3	
	**Snack**	Apple/pear/banana	100	
**Control diet**	**Breakfast**	Mix of yoghurt and quark	30	
		Corn flakes	30	
		Orange juice	50	
		White bread	20	
		Sandwich spread based on mostly butter	20	
		Cheese	20	
	**Main meal**			
	Carbohydrate	Potatoes	32	
		White rice	18	
	Protein	Lamb	6	
		Beef	29	Meat balls including both beef and pork (8 meals)
		Chicken	15	
		Egg	6	
	Greens	Chickpeas	6	
		Tomatoes	21	Canned/purée
		Beetroots	6	

			Number of meals	Comment
		Haricots verts	3	
		Mushroom	22	
		Bamboo shoots	4	
		Corn	4	
		Pineapple	4	
		Carrot	3	
		Onion/garlic	50	
	Sauce	Yoghurt	6	
		Cream	26	
		Coconut milk/-cream	10	
		Smetana	6	
		Cheese	3	
	Other	Flavoring: Broth, jelly, vinegar, starch, spices etc.	50	
		Butter	20	
		Rapeseed oil	18	
		Olive oil	6	
		Other oils	13	
**Snack**		Quark	20	
		Protein pudding	10	
		Protein bar	20	
