# Supplementary material for: Dietary biomarkers and food records indicate compliance to study diets in the ADIRA (Anti-inflammatory Diet In Rheumatoid Arthritis) trial
Source: Front Nutr. 2023 Jun 22;10:1209787. doi: 10.3389/fnut.2023.1209787 (PMC10325030; doi:10.3389/fnut.2023.1209787)
Supplement: Supplementary file 3 [file Data_Sheet_3.PDF]

frontiers

Supplementary Material

SUPPLEMENTARY TABLE 2 | Food items included and excluded when manually extracting the daily intake of fruit, berries and vegetables, seafood, and red meat from the analyzed food records.

	Included	Excluded
**Fruit, berries and vegetables**	Fresh/frozen/dried fruit and berries	Jam and jelly
	Fresh/frozen vegetables and root vegetables	Broth
	Canned/pureed tomatoes	Quorn
	Tomatoes in ketchup/other tomato-based sauces	Potatoes
	Mushrooms	Fruit/berries in yoghurt/milkshake
	Onions	Fresh herbs
	Olives and other canned vegetables	Nuts (incl. peanuts)
	Legumes	Fruit/berries in desserts
	Juice from lemon and lime	Juice and other fruit-based beverages
**Seafood**	Fresh/frozen/smoked fish, shellfish, and roe	Broth
**Red meat**	Fresh/frozen/smoked pork, beef, and lamb	Poultry
	Fresh/frozen/smoked/dried game meat	Broth
