# Supplementary material for: Dietary biomarkers and food records indicate compliance to study diets in the ADIRA (Anti-inflammatory Diet In Rheumatoid Arthritis) trial
Source: Front Nutr. 2023 Jun 22;10:1209787. doi: 10.3389/fnut.2023.1209787 (PMC10325030; doi:10.3389/fnut.2023.1209787)
Supplement: Supplementary file 4 [file Data_Sheet_4.PDF]

frontiers

Supplementary Material

SUPPLEMENTARY TABLE 3 | Plasma- and serum dietary biomarkers and reported dietary intake[1] in participants completing at least one diet period in the randomized crossover trial ADIRA (Anti-inflammatory Diet In Rheumatoid Arthritis).

	Intervention diet[2]			Control diet[3]		
**Plasma/ Serum**	**Pre (n=46)**	**Post (n=45)**	**Δ (n=45)[4]**	**Pre (n=47)**	**Post (n=46)**	**Δ (n=46)[4]**
Total AR	45.3 (25.5, 74.9)	45.4 (30.8, 93.3)	7.2 (-16.7, 44.2)	47.1 (22.1, 80.0)	46.2 (18.2, 77.8)	-2.3 (-25.4, 21.7)
C17:0/C21:0	0.23 (0.13, 0.36)	0.16 (0.08, 0.30)	-0.05 (-0.13, 0.08)	0.23 (0.13, 0.35)	0.22 (0.16, 0.33)	-0.01 (-0.09, 0.07)
AR homologues (nmol/L)						
C17:0	3.7 (1.7, 7.6)	2.9 (1.3, 6.6)	-0.3 (-2.4, 2.0)	3.1 (1.9, 7.0)	3.2 (1.5, 6.5)	0.3 (-2.4, 1.9)
C19:0	11.3 (6.5, 21.4)	10.7 (6.8, 20.1)	1.6 (-3.7, 5.3)	11.5 (6.2, 20.9)	12.9 (5.8, 19.0)	1.1 (-6.1, 7.6)
C21:0	16.9 (9.4, 27.9)	18.0 (11.1, 34.9)	4.5 (-3.9, 15.6)	14.8 (8.7, 30.9)	17.0 (6.5, 23.8)	-1.0 (-11.5, 8.9)
C23:0	7.4 (3.9, 11.1)	8.1 (4.1, 16.8)	1.6 (-2.3, 9.8)	7.4 (3.1, 12.5)	6.0 (2.4, 11.7)	-0.8 (-5.9, 3.9)
C25:0	5.4 (2.6, 8.2)	5.2 (2.8, 11.6)	0.4 (-3.6, 5.5)	4.8 (2.1, 11.2)	4.0 (1.5, 9.1)	-0.1 (-2.6, 3.0)
Carotenoids (µmol/L)						
Lutein + zeaxanthin	0.33 (0.21, 0.43)	0.38 (0.26, 0.49)	0.04 (-0.01, 0.10)	0.32 (0.21, 0.41)	0.35 (0.25, 0.48)	0.03 (-0.02, 0.10)
β-cryptoxanthin	0.17 (0.13, 0.30)	0.18 (0.12, 0.34)	-0.01 (-0.07, 0.02)	0.22 (0.12, 0.30)	0.25 (0.16, 0.40)	0.04 (-0.01, 0.12)
Lycopene	0.46 (0.30, 0.74)	0.51 (0.34, 0.66)	0.00 (-0.10, 0.12)	0.50 (0.31, 0.70)	0.50 (0.35, 0.86)	0.08 (-0.04, 0.20)
α-*carotene*	0.09 (0.05, 0.17)	0.09 (0.05, 0.14)	-0.01 (-0.04, 0.01)	0.08 (0.05, 0.15)	0.11 (0.06, 0.16)	0.00 (-0.02, 0.04)
β-*carotene*	0.53 (0.33, 0.92)	0.50 (0.33, 0.80)	-0.03 (-0.12, 0.05)	0.51 (0.36, 0.76)	0.52 (0.35, 0.82)	-0.01 (-0.13, 0.08)

	Intervention diet[2]			Control diet[3]		
**Plasma/ Serum**	**Pre (n=46)**	**Post (n=45)**	**Δ (n=45)[4]**	**Pre (n=47)**	**Post (n=46)**	**Δ (n=46)[4]**
Total carotenoids	1.80 (1.18, 2.42)	1.89 (1.32, 2.35)	-0.02 (-0.24, 0.18)	1.71 (1.27, 2.31)	1.93 (1.36, 2.61)	0.18 (-0.16, 0.47)
Total carotenoids excl. lycopene	1.10 (0.78, 1.83)	1.33 (0.75, 1.80)	-0.02 (-0.15, 0.18)	1.14 (0.87, 1.54)	1.35 (0.97, 1.75)	0.14 (-0.13, 0.28)
FA (% of total FA)						
LA (18:2, n-6)	26.9 (23.7, 29.9)	28.3 (23.8, 31.4)	1.29 (-0.15, 3.41)	27.3 (23.1, 29.9)	26.6 (22.5, 29.6)	-0.18 (-2.30, 0.94)
ALA (18:3, n-3)	0.87 (0.76, 1.04)	0.88 (0.73, 1.05)	-0.02 (-0.10, 0.14)	0.85 (0.70, 1.02)	0.85 (0.76, 1.03)	-0.03 (-0.10, 0.11)
EPA (20:5, n-3)	1.15 (0.97, 1.61)	1.49 (1.12, 1.90)	0.25 (-0.12, 0.51)	1.28 (0.92, 1.67)	1.23 (0.94, 1.48)	-0.02 (-0.27, 0.08)
DHA (22:6, n-3)	2.43 (2.00, 2.97)	2.80 (2.50, 3.36)	0.36 (0.08, 0.86)	2.44 (1.94, 3.01)	2.22 (1.84, 2.71)	-0.25 (-0.61, 0.02)
DPA (22:5, n-3)	0.56 (0.46, 0.59)	0.51 (0.48, 0.60)	-0.01 (-0.05, 0.05)	0.52 (0.46, 0.63)	0.55 (0.48, 0.63)	0.02 (-0.02, 0.06)
**Dietary intake (g/day)**	**Pre (n=46)**	**Post (n=43)**	**Δ (n=43)[4]**	**Pre (n=47)**	**Post (n=42)**	**Δ (n=42)[4]**
Whole grain	28 (14, 43)	33 (24, 44)	10 (-7, 22)	29 (15, 41)	7 (1, 25)	-17 (-28, -1)
FBV	269 (189, 405)	492 (389, 586)	178 (66, 305)	309 (188, 371)	188 (117, 328)	-87 (-168, 1)
FBV + JFB	339 (222, 435)	516 (414, 638)	196 (99, 295)	324 (234, 446)	377 (309, 445)	46 (-54, 137)
Seafood	22 (0, 66)	90 (53, 124)	53 (8, 89)	41 (17, 78)	5 (0, 44)	-31 (-50, 0)
Red meat	49 (19, 76)	13 (0, 39)	-34 (-53, 0)	46 (25, 92)	79 (53, 109)	31 (-7, 57)

Values refer to median (IQR).
[1]*Assessed using 3-day food records*
[2]*A proposed anti-inflammatory diet rich in fatty fish, whole grain, fruit, and vegetables*
[3]*A diet nutritionally alike a Swedish diet*
[4]*Calculated as the median (IQR) of all participants' individual difference between post- and pre values of each diet period.*
ALA, α-linolenic acid; AR, Alkylresorcinols; DHA, Docosahexaenoic acid; DPA, Docosapentaenoic acid; EPA, eicosapentaenoic acid; FA, Fatty acids; FBV, Fruit, berries and vegetables; JFB, Juice and fruit-based beverages; LA, Linoleic acid
